# Recent Increase in Winter Hazy Days over Central India and the Arabian Sea

**DOI:** 10.1038/s41598-019-53630-3

**Published:** 2019-11-22

**Authors:** Abin Thomas, Chandan Sarangi, Vijay P. Kanawade

**Affiliations:** 1 Centre for Earth, Ocean and Atmospheric Sciences, University of Hyderabad, Hyderabad, Telangana 500046 India; 2 Pacific Northwest National Laboratory, Richland, Washington 99352 USA

**Keywords:** Atmospheric science, Climate change

## Abstract

Indian subcontinent is greatly vulnerable to air pollution, especially during the winter season. Here, we use 15 years (2003–2017) of satellite and model reanalysis datasets over India and adjoining Seas to estimate the trend in hazy days (i.e. days with high aerosol loading) during the dry winter season (November to February). The number of hazy days is increasing at the rate of ~2.6 days per year over Central India. Interestingly, this is higher than over the Indo-Gangetic Plain (~1.7 days/year), a well known global hotspot of particulate pollution. Consistent increasing trends in absorbing aerosols are also visible in the recent years. As a result, the estimated atmospheric warming trends over Central India are two-fold higher than that over Indo-Gangetic Plain. This anomalous increment in hazy days over Central India is associated with the relatively higher increase in biomass burning over the region. Moreover, the trend in aerosol loading over the Arabian Sea, which is located downwind to Central India, is also higher than that over the Bay of Bengal during the dry winter season. Our findings not only draw attention to the rapid deteriorating air quality over Central India, but also underline the significance of increasing biomass burning under the recent climate change.

## Introduction

Aerosols are ubiquitous in the atmosphere. While natural aerosols constitute the largest fraction of global aerosol burden, regional hotspots of high aerosol loading coincide with regions of high population density, urbanization and industrialization, or biomass burning. The long-term measurements of aerosols^1–3^, observational campaigns^4–6^, and remote sensing from ground and space^7,8^ have provided requisite datasets to improve our understanding of the physical, optical and chemical properties of aerosols. The extensive observational and modelling efforts over the last two decades have aided remarkably to advance our knowledge of aerosols influence of the climate^9^. For instance, the cooling effect from increasing aerosols has masked about one-third of the increasing greenhouse warming over the past half-century^10^. However, the lack of understanding of the variability of aerosols and their effects at regional scale contribute to the existing large uncertainty in aerosol feedbacks in future climate predictions.

The Indian subcontinent and adjoining Seas experience a tropical and sub-tropical climate and have been a focus of the study for aerosols over the last two decades. Observational campaigns (e.g. Indian Space Research Organization-Geosphere Biosphere Programme, ISRO-GBP; Indian Ocean Experiment, INDOEX; Arabian Sea Monsoon Experiment, ARMEX) have shown large aerosol negative radiative forcing at the surface and relatively large atmospheric warming than top of the atmosphere (TOA)^11–13^. The wintertime hazardous air pollution scenarios over the densely populated regions of India have recently received the utmost scientific attention. The winter season is characterized by a shallower boundary layer, lower wind speed and low precipitation, leading to the accumulation of aerosols near the surface^2,14,15^. Along with the obvious health impacts, studies have shown that the high aerosol loading in winter can reduce radiation reaching to the surface by about 25%, thereby decreasing crop yield^16–18^. Nonetheless, aerosols act as cloud condensation nuclei and affect cloud formation and rainfall^19^. Therefore, accurate knowledge of trend in aerosols over India during the winter season is extremely essential for reducing uncertainty in future climate, health, and economic predictions.

In India, several recent studies have used satellite and ground based measurements spanning over decade to find the trend in aerosol loading over Indian region^2,20–28^. *Dey and Girolamo*^20^ and *Dey et al*.^26^, found a significant rise in anthropogenic aerosols over the Indian subcontinent during 2000–2010 using MISR aerosol optical depth (AOD) data. These studies highlighted that the rural areas of IGP are more polluted than that of urban cities in the peninsular India. *Kaskaoutis et al*.^22^, found an increasing trend in AOD over Kanpur (located in IGP), especially during the post-monsoon and winter seasons based on Aerosol Robotic Network (AERONET) AOD data from 2001 to 2010. *Hsu et al*.^25^, also found an increasing trend in fine mode anthropogenic aerosols over North India and Bay of Bengal (BoB), particularly during the dry winter and post-monsoon seasons using SeaWiFS measurement from 1997 to 2010. *Ramachandran et al*.^23^, attributed the incremental trend in aerosols over New Delhi to anthropogenic aerosols and over Northeast India to increase in forest fire and biomass burning emissions. *Babu et al*.^2^, showed an increasing trend in anthropogenic contribution to total aerosol loading during the dry winter season. *Moorthy et al*.^21^, *also* found an increasing trend in aerosol loading in the current decade than its value in 1985. *Srivasthava*^24^ highlighted that the more than 70% of the Indian subcontinent shows a positive trend in AOD from 2 to 6% during the winter and pre-monsoon seasons, with a trend of >6% over BoB. *Kumar et al*.^27^, recently found a relatively high aerosol loading over IGP as compared to other parts of India, but a statistically insignificant increasing trend of 0.002 AOD/year using MODIS-TERRA and nine ground-based stations data. They also observed a strong seasonality in aerosol loading with the dominance of fine mode aerosols over IGP, especially during the dry winter season. Most of these previous studies highlight the increasing trend of aerosol loading over highly polluted IGP and northern BoB in last two decades due to anthropogenic emissions.

Using 15 years of satellite (MODIS and OMI) observations and reanalysis (MERRA-2) data products, we illustrate that aerosol loading over Central India and the Arabian Sea during the dry winter season is increasing at a greater rate than that over IGP and the BoB in the recent years. We focused our analyses for the dry winter season (November to February) since the number of hazy days is highest during the season. A hazy day is referred to as the day with high aerosol loading (i.e. AOD greater than 66^th^ percentile value over a location).

## Results and Discussion

### Trend in the number of hazy days

**Figure 1:**
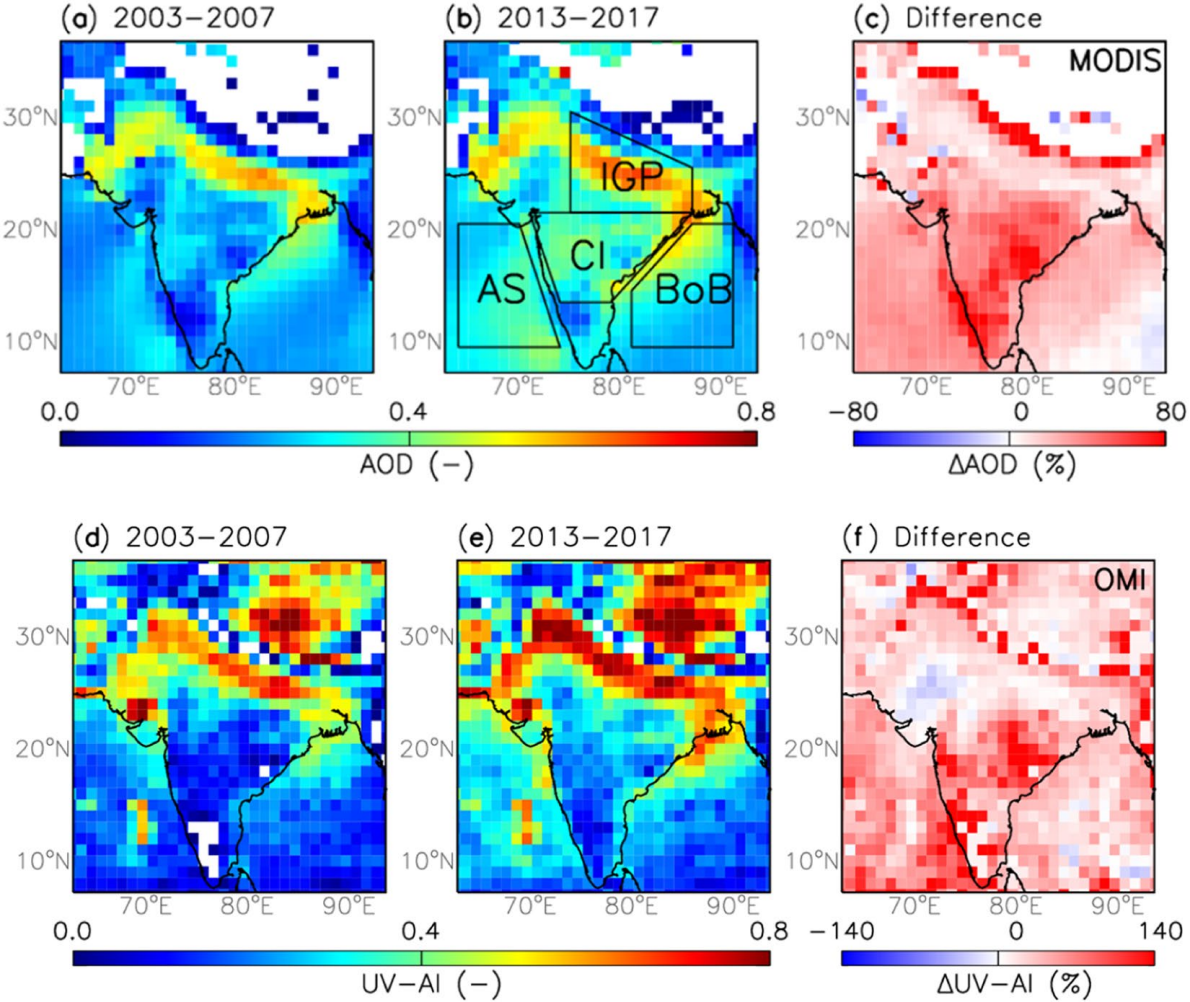
Averaged spatial distribution of MODIS AOD for November through February over the time periods (**a**) 2003–2007 (past years), (**b**) 2013–2017 (recent years) and (**c**) the percentage difference between the recent and the past years. The study regions are bounded by solid black line shown in (**b**); IGP: Indo-Gangetic Plain, AS: Arabian Sea, CI: Central India and BoB: Bay of Bengal. Averaged spatial distribution of OMI UV-AI for November through February over the time periods (**d**) 2003–2007, (**e**) 2013–2017, and (**f**) the percentage difference between the recent and the past years.

The entire time period (2003–2017) is split into two sub-periods; past years (2003–2007) and recent years (2013–2017) to highlight changes in aerosol loading in the current decade compared to that of the previous decade. Figure 1 shows averaged spatial distribution of MODIS columnar AOD and OMI UV aerosol index (UV-AI) over India and adjoining Seas for the past and the recent years. The increased aerosol loading in the recent years (2013–2017) is clearly evident, analogous to several previous studies^2,20–25,27^. The rate of change in the aerosol loading between the past and the recent years is distinctive regionally due to the variability in aerosol emission rates and latitudinal diverse climatic conditions. OMI retrieved UV-AI, which is a measure of UV-absorbing aerosol particles such as soot/smoke and mineral dust, show similar distinctive enhancement as that of AOD over Central India. This suggests the incremental dominance of absorbing aerosols over Central India in the recent years.

**Figure 2:**
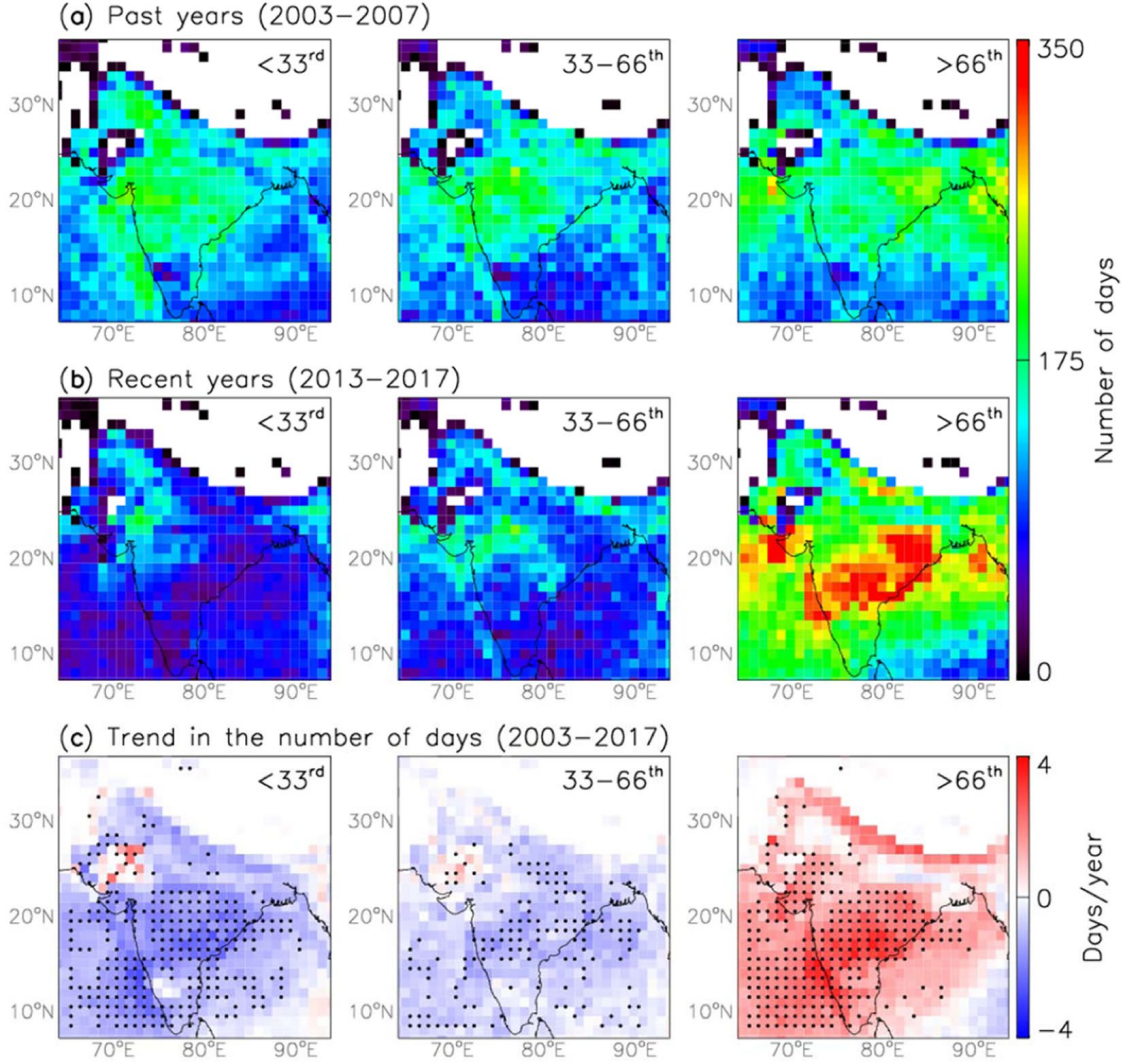
Trend in the number of days with aerosol loading from low to high regimes. (**a**) The number of days with low (<33^rd^ percentile value of AOD), medium (33–66^th^) and high (>66^th^) aerosol loading for November-February of 2003–2007 (**b**) same as (**a**) except that for 2013–2017. (**c**) Trend in the number of days with <33^rd^, 33–66^th^ and >66^th^ percentile values of AOD for November–February of 2003–2017. Black dots indicate statistical significance using Student’s t-test at a confidence interval of 95%.

We first compare the number of days from low to high aerosol loading between the past and the recent years. In order to do this, the daily AOD observations are segregated into three percentile bins: AOD less than 33^rd^ percentile, between the value 33^rd ^and 66^th^ percentile, and greater than 66^th^ percentile values over each 1° × 1° grid based on the AOD values for November through February of the year 2003. The bins are identified as three distinct aerosol loading regimes; low (<33^rd^ percentile), medium (33–66^th^ percentiles), and high (>66^th^ percentile). Then, the number of days for each of these regimes is counted during the past (Fig. 2(a)) and the recent (Fig. 2(b)) time periods. The number of days with low and medium aerosol loading has reduced in the recent years as compared to the past years (Fig. 2(a,b)). But, the days with high aerosol loading (>66^th^ percentile) have increased in the recent years over India and adjoining Seas (Fig. 2(a,b)). Interestingly, the rate of increase in the number of high aerosol loading (i.e. hazy) days over CI is higher (~2.6 days/season) than over IGP (~1.8 days/season) (Fig. S1). This rate is also higher over the AS (~1.9 days/season) than over the BoB (~1.1 days/season). Noticeably, all the four regions have about two-fold high aerosol loading days in the year 2017 as compared to the year 2003 (Fig. S1).This indicates that the aerosol burden over India is typically shifted to higher values in the recent decade relative to the previous decade. It should be noted that MODIS retrieved AOD over IGP in the dry winter season is often plagued under foggy conditions, but we have removed AOD > 1.0 to avoid fog/cloud contaminated AOD retrievals in our analysis^29,30^. Wintertime fog thickens overnight and on till about an hour after sunrise. Then, it gradually disperse or thin out by noontime via effective ventilation^31^ and since AQUA instrument onboard MODIS has overpass over India at about 1:30 pm local time, chances of fog to influence the results are negligible.

Further, the trend in the number of days for each of the percentile regime is calculated from 2003 to 2017 (Fig. 2(c)). The trend in the number of days with low to medium aerosol loading days is found to be negative over India and adjoining Seas. But, the trend in the number of days with high aerosol loading days is found to be positive, with significantly positive values over CI and the AS as compared to IGP, analogous to our finding from Fig. 2(b). Previous studies have reported increasing aerosol loading trend over India using both the ground-based^2,21,22,28^ and satellite observations^1,20,23,24,32^. But, we find that the increasing aerosol loading trend is more pronounced over CI and the AS, with >2 days per season over the time period from 2003 to 2017. *Moorthy et al*.^33^, also revealed that the rate of increase in columnar AOD was more rapid (~4%) during the time period from 2000 to 2011 compared to the previous decade and that Central Peninsular India showed the highest increasing trend (3.63%/year) than the Southern Peninsular and Northern India, with higher rates during the winter season as compared to other seasons. A recent study also showed less significant increasing trends in AOD over IGP over last decade^27^.

**Figure 3:**
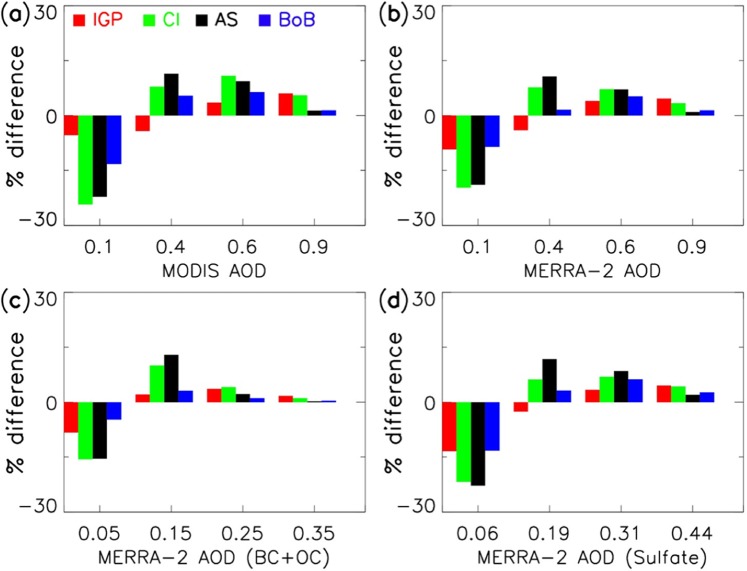
The percentage difference in the frequency distribution of (**a**) MODIS AOD, (**b**) MERRA-2 AOD, (**c**) MERRA-2 (BC + OC) AOD and (**d**) MERRA-2 sulfate AOD between the past and the recent years over different study regions.

The difference in the percentage frequency distribution for MODIS AOD between the past and the recent years shows a decreasing frequency of low aerosol loading (AOD bin of 0.1), whereas it shows an increasing frequency of medium to high aerosol loading (AOD bin >0.4) (Fig. 3(a)) over all study regions. We also find that MERRA-2 reanalysis data is able to reproduce similar intensity-frequency variation in the AOD (Fig. 3(b)). In order to examine the changes in various aerosol species associated with the observed increasing trend in total aerosol loading, we calculate the percentage frequency distribution for species-wise AOD separately for the past and the recent years for different regions of India (Fig. S2). The differences in the percentage frequency distribution for these variables between the past and the recent years are then plotted in Fig. 3(c,d). The BC and OC AOD is clubbed together as they are co-emitted from various local sources, both anthropogenic aerosols and biomass burning. The natural emissions like sea salt (SS) and dust AOD, which are mostly transported into CI and IGP domain, are also shown in Fig. S3. Intensity-frequency variability similar to that seen for composite AOD (Fig. 3(a,b) is present for BC + OC and sulfate (Fig. 3(c,d)) species over both IGP and CI regions. Interestingly, similar changes are not seen for the case of transported species i.e. SS and dust AOD which indicates that the change in total aerosol loading may be attributed to increase in the local emissions. Moreover, the intensity-frequency variation is more prominent over CI and the AS compared to that over IGP. Further, the differences in the vertical aerosol mixing ratio profiles illustrate significant enhancement in BC and OC from the surface to 700hPa (~3.1 km) which imply near-surface sources (Fig. S5). But, the anomalous enhancements in BC and OC mixing ratios are higher over CI in the recent years than over the IGP.

Interestingly, the intensity-frequency changes in AOD observed over the AS region is more prominent compared to the BoB region. In addition, the anthropogenic species over the AS, particularly BC, OC, and sulfate, are found to be three times higher than SS aerosols during the winter season (Figs. 3 and S3). The mean wind circulation clearly illustrates predominant northeasterly flow over the continental region mostly flowing into the AS i.e. the AS is located in the downwind regions of CI (Fig. S4). Thus, the BC, OC, and sulfate aerosols over the AS are probably transported from local anthropogenic emissions over the CI. This also explains the high percentage change in aerosol loading over the AS than the BoB in the recent years (Fig. 3). This suggests that the increase in continental aerosol emissions has profound impact on the aerosol loading over the adjacent marine regions. This finding is different from previous investigators as those studies indicated that the aerosol loading over the BoB is higher than over the AS, and that the relative contribution of anthropogenic aerosol mass tend to be higher over the BoB^34–37^. It could be noted that most of these studies used data prior to the year 2012, except the recent study by Srivastava^24^, further highlighting the significance of our finding on aerosol perturbations in the recent years (2013–2017).

**Figure 4:**
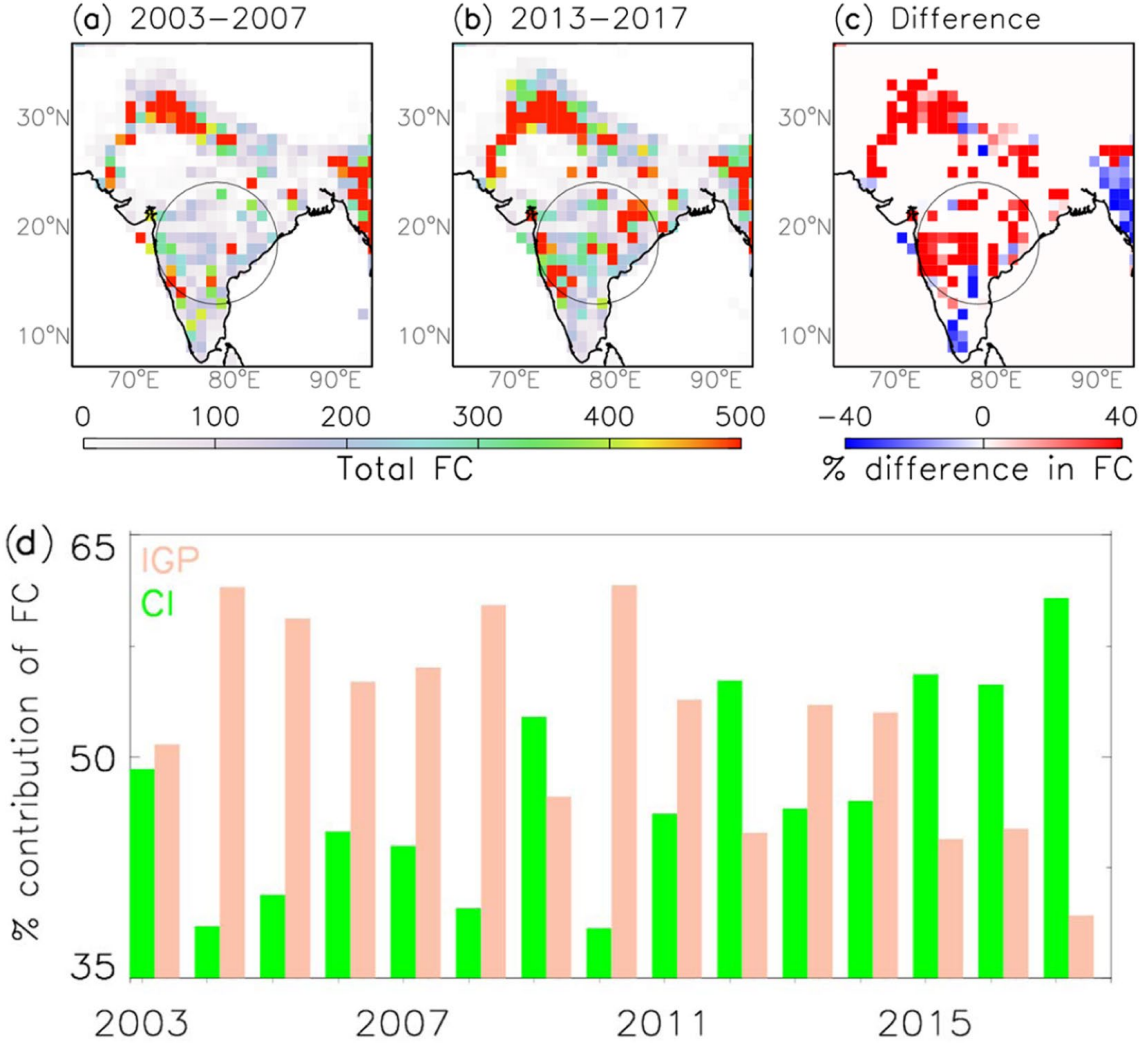
(**a**) Spatial map of 1° × 1° gridded total fire counts (FC) for November-February of 2003–2007 (past years). (**b**) Same as (**a**) except that for 2013–2017 (recent years). (**c**) The difference between the past and the recent years. (**d**) The change in percentage contribution of fire counts over CI and IGP for November-February of 2003 to 2017.

Rapid urbanization in developing nations like India is generally the primary source of the overall aerosol burden^38,39^. For example, out of the ten most populous metropolitan areas in India, five of them lie within CI region (Bangalore, Hyderabad, Mumbai, Nagpur and Pune) and these cities are known for rapid change in the land use and land cover over the last decade^40^. It should be noted that forests, shrubs, and cropland contributes to a large fraction of the land cover over Central India. As a result, the biomass burning activities peak within the two central states (Madhya Pradesh and Maharashtra), accounting for about 36% of the total fire counts in India^41^. Besides, the Eastern Ghats in Central Eastern India is a dense active fire hotspot, owing to shifting cultivation practices and clearing of mixed deciduous forest in the late winter season^42,43^. Therefore, biomass burning (e.g. forest fires, crop residue burning, trash/wood burning), which is a major sources of aerosol loading over India^44^, can contribute significantly to this anomalous enhancement in AOD in the recent decade. Figure 4(a–c) presents spatial map of 1° × 1° gridded total fire counts over India during the past and the recent years and difference between them. The spatio-temporal variability in fire counts over CI (Fig. 4(a,b)) is consistent with the spatial variability in aerosol loading and AI (Fig. 1). Further, the monthly fire count difference (Fig. S6) correlates spatially with the monthly trend in the number of days with high aerosol loading (Fig. S7), particularly over CI. We have then counted the number of fire hotspots for each year over IGP and CI, and calculated the percentage contribution of fire counts from both the regions. Note that the number of 1° × 1° gridded pixels in IGP and CI are equal (n = 75). The relative percentage of fire counts over CI and IGP are plotted in Fig. 4(d). The percentage contribution of fire counts over CI has increased at a rate of 20% from 2003 to 2017 indicating that the fire counts over CI is higher than that over IGP in the recent years. Thus, the greater rate of enhancement in high aerosol loading days over CI in the recent years (Fig. 2(b)) can be associated with relatively large enhancement in fire emissions over CI (Fig. 4(c)) compared to IGP. Moreover, a recent study show that a large fraction of aerosols from biomass burning activities over northern western states can also extend over parts of Central India^45^ as well as northwestern parts of Bay of Bengal^34^. Therefore, the observed enhancement in the recent aerosol loading over CI can be a combined result of rapid urbanization, enhanced localized fire emissions, and long-range transport of aerosols from the IGP region.

### Change in aerosol direct radiative forcing

Aerosol direct radiative forcing counteracts, in part, the warming due to greenhouse gases^10,46^, but the effects may vary temporally and spatially. High aerosol direct radiative forcing is usually found over India due to the combined effect of dust, smoke and other aerosols^32^. In this section, we present the change in the ADRF at the top of the atmosphere, on the atmosphere and at the surface using MERRA-2 data. Figures S8–S10 presents the averaged spatial distribution of the ADRF at the TOA, on the atmosphere and at the surface, respectively, during the past and the recent years and the difference between these two time periods. *Nair et al*.^47^, have shown that aerosol forcing at the TOA is modulated mostly by anthropogenic aerosols. It is clear that the increasing trends in aerosol loading over India and adjoining Seas, especially off the eastern and western coasts, led to increased cooling at the TOA in recent years (Fig. S8). *Kaskaoutis et al*.^34^, have observed significant fraction of soot aerosols over northwestern BoB during the winter. In fact, the aerosol-induced atmospheric forcing efficiency was found higher for the BoB (31 W/m^2^) as compared to the AS (18 W/m^2^)^48^, but we find that ADRF on the atmosphere is higher over the AS in the recent years. It is apparent that there is increased atmospheric warming (Fig. S8), increased surface cooling (Fig. S9), and increased TOA cooling (Fig. S10) over India and adjoining Seas owing to increased aerosol loading (Figs. 1 and 2) in the recent years.

To illustrate how the forcing has changed over the study period, we calculate ADRF for each region separately (Fig. S11). The overall atmospheric (positive) and surface (negative) forcing is highest over IGP compared to CI and adjoining Seas (Fig. S11). But, what is interesting is that the difference between the recent and the past years in atmospheric warming over CI (4.50 W/m^2^) overtakes IGP (2.01 W/m^2^) (Table S2). Concurrently, the mean difference in ADRF on the atmosphere between the recent and the past years is higher over the AS (3.67 W/m^2^) than that of the BoB (0.48 W/m^2^), as opposed to previous studies^48,49^. This suggests that aerosols exerted as much as seven times more atmospheric warming over the AS than the BoB in recent years. The regionally averaged ADRF on the surface and at the TOA are also higher over the AS than the BoB (Table S2), contradicting to the previous study which showed larger values over the BoB for February 2003^50^. These findings imply that the increasing fire activity over CI has altered the forcing at much expected level over CI and downwind AS than the polluted IGP. The enhanced atmospheric heating and surface cooling over CI and the AS can lead to increase in lower tropospheric stability. The stable atmospheric condition would then favor more accumulation of aerosols close to the surface and can further accelerate occurrence of hazy days (Fig. S12), and thus, creating a positive feedback mechanism. Nonetheless, the increase in LTS can itself be influenced by the increase in aerosol-induced atmospheric warming^51^. Therefore, this aerosol-LTS coupling can induce a positive feedback cycle on aerosol accumulation in the boundary layer and enhance aerosol loading over CI and the AS. Moreover, enhanced atmospheric stability may also enhance low-level cloud amount over the AS and the BoB, leading to a negative feedback on the climate system in a warming anthropogenic future.

## Conclusions

Indian subcontinent, one of the world’s fastest growing regions in terms of urbanization and population, is greatly vulnerable to particulate pollution. The winter time hazy scenarios and their radiation feedbacks exert a profound impact on the weather and climate. Using 15 years (2003–2017) of satellite and reanalysis datasets, this study investigates the trend in the number of hazy days (i.e. days with high aerosol loading) and the aerosol-induced direct radiation feedbacks on the surface-atmosphere system over India and adjoining Seas for the dry winter season (November–February).

The major findings of this study are as follows;

1. Overall, aerosol loading over India and adjoining Seas is rapidly increasing in the recent years.
2. The number of hazy days are increasing at the rate of about 2 days per year over India, with a higher rate over CI (~2.6 days/year) than that of over IGP (~1.7 days/year).
3. Since the AS is located downwind to the CI, the number of hazy days over the AS is also higher than that over the BoB during the dry winter season.
4. Collocated similar enhancements in UV aerosol index as that of AOD suggests the dominance of absorbing aerosols over Central India in the recent years.
5. Consequently, aerosol-induced atmospheric warming (4.50 W/m^2^) and surface cooling (−9.44 W/m^2^) due to aerosol direct radiative forcing is highest over CI as compared to other study regions in the recent years. The enhanced atmospheric warming over CI is about two-fold to that of over IGP (2.01 W/m^2^).
6. The higher aerosol loading over CI is attributed to the recent increase in biomass burning activities over the region.
7. Surprisingly, aerosols exerted as much as seven times more atmospheric warming over the AS in the recent years than over the BoB. This contradicts to majority of previous studies which showed higher atmospheric warming over the BoB than the AS during the winter season. This finding is substantiated by the mean changes in wind speed and lower tropospheric stability over CI and the AS.

Although the high aerosol loading is observed over IGP than other regions, our study reveals that aerosol loading over CI has escalated greatly in the recent years. Thus, our findings provide new insights to better constrain aerosols role in the climate over Indian subcontinent.

## Methods

In this study, we have used the geographical region bounded by latitude, 7–38°N, and longitude, 66–94°E. This region further divided into four sub-regions viz., Indo-Gangetic Plain (IGP), Central India (CI), Arabian Sea (AS) and Bay of Bengal (BoB) (Fig. 1b). The columnar composite AOD from Moderate-resolution Imaging Spectroradiometer (MODIS) onboard AQUA is used. The modeled aerosol species concentration (sulfate, BC, OC, sea salt, and dust) in terms of their optical depth and radiation fluxes are used from the Modern-Era Retrospective Analysis for Research and Application - version 2 (MERRA-2). These observations and modelled values at daily resolution over the time period from 2003 to 2017 for the time period from October through February are used. Also the composition of aerosols and the meteorological conditions have insignificant monthly variability during the dry winter season^18^. Table S1 summarizes data products and their temporal and spatial resolution.

### MODIS

The Moderate-resolution Imaging Spectroradiometer sensor onboard polar orbiting Earth Observing System (EOS) satellite AQUA flies at an altitude of 705 km, with a swath width of 2330 km and equator crossing at 13:30 local time. It measures the reflected solar radiance and terrestrial emission in a wavelength band ranging from 0.41–14.4 μm divided into 36 channels, categorized with horizontal resolutions varying between 0.25 and 1 square kilometer^52^. Here, we have used the level3 Dark Target and Deep Blue combined AOD of collection 6.1 at 0.55 µm at a grid resolution of 1° × 1° ^53–55^. The estimated maximum uncertainty is approximately ±0.05 × AOD over Oceans and ±0.15 × AOD over continents. The technical details, algorithm and validation details can be found in *Remer et al*.^52^. The collection 6 MODIS active fire location product at 1 km resolution was also used as proxy for biomass burning hot spots. The product utilizes thermal anomalies in infrared wavelengths (4 and 11 μm)^56^.

### OMI

In addition to MODIS, AURA Ozone Monitoring Instrument (OMI) daily UV aerosol index (UV-AI) is used (OMAERUVd.003) in this analysis. AURA OMI is a nadir-viewing spectrometer onboard NASA’s Aura satellite. It measures direct and backscattered solar radiation in the UV-visible range from 264 to 504 nm. The retrieval technique and validation is given in *Bucsela et al*.^57^. UV-AI is based on a spectral contrast method in a UV region where the ozone absorption is negligible. Positive values of UV-AI indicate absorbing aerosols (smoke and dust) whereas near-zero or negative values indicate non-absorbing aerosols (sulfate and sea salt) and clouds^58^. While, UV-AI is good indicator of absorbing aerosols, its value is dependent on the smoke plume altitude^59^. The OMI retrieval algorithm for aerosol detection has been validated with ground-based measurements^60^. OMI UV-AI is available from the NASA Goddard Earth Sciences, Data and Information Services Center (GES DISC; http://disc.sci.gsfc.nasa.gov).

### MERRA-2

The Modern-Era Retrospective Analysis for Research and Application - version 2^61^ uses the Goddard Earth Observing System-5 (GEOS-5) atmospheric general circulation model^62^ that is coupled with the Goddard Global Ozone Chemistry Aerosol Radiation and Transport model (GOCART)^63^. The GOCART model simulates AOD for five major aerosol species like OC, BC, sea salt, dust and sulfate using multi-satellite based (MODIS,AVHRR and MISR) and ground-based (AERONET) AOD data. GEOS-5 model provides the data from 1980 to present in hourly and monthly gridded data with the resolution of 0.5° × 0.625° in latitude and longitude from the surface to the top layer of 0.01 hPa with 72 vertical levels^64^. The MERRA-2 simulated AOD compares well with ground-based and satellite measurements globally^64,65^. It is freely available from NASA Goddard Earth Sciences (GES) Data and Information Services Center (DISC) https://disc.gsfc.nasa.gov/. In order to calculate clear-sky aerosol direct radiative forcing (ADRF) from the radiative fluxes from MERRA-2 data^65^, the difference between the radiation flux under clear sky condition in the presence of aerosol and without aerosol is calculated. The hourly variables; SWGNTCLR (surface net downward shortwave flux assuming clear-sky), SWGNTCLRCLN (surface net downward shortwave flux assuming clear-sky and no aerosol), LWGNTCLR (surface net downward longwave flux assuming clear sky) and LWGNTCLRCLN (surface net downward longwave flux assuming clear-sky and no aerosol) are used to calculate ADRF at surface (ADRF_SURF_). Concurrently, ADRF at the top of the atmosphere (ADRF_TOA_) is calculated from hourly variables, SWTNTCLR (TOA net downward shortwave flux assuming clear sky), SWTNTCLRCLN (TOA net downward shortwave flux assuming clear-sky and no aerosol), LWTUPCLR (upwelling longwave flux at TOA assuming clear-sky) and LWTUPCLRCLN (upwelling longwave flux at TOA assuming clear-sky and no aerosol). These radiation variables can be found in the MERRA-2 product, tavg1_2d_rad_Nx. Overall, MERRA-2 simulated radiative fluxes agrees well with CERES EBAF Edition 2.8 satellite product over 2001–2015^66^. MERRA-2 simulated both shortwave and longwave radiative fluxes are used to derive the total aerosol direct radiative forcing (ARDF). ADRF at the surface (*ADRF*_SUR_) and at top of the atmosphere *(ADRF*_TOA_) are calculated by the following formula.

$$\begin{array}{c}{{\rm{ADRF}}}_{{\rm{SUR}}}=({\rm{SWGNTCLR}}+{\rm{LWGNTCLR}})-({\rm{SWGNTCLRCLN}}+{\rm{LWGNTCLRCLN}})\\ {{\rm{ADRF}}}_{{\rm{TOA}}}=({\rm{SWTNTCLR}}+{\rm{LWTUPCLR}})-({\rm{SWTNTCLRCLN}}+{\rm{LWTUPCLRCLN}})\end{array}$$

The ADRF on the atmosphere, which indicates the energy trapped by all aerosols in the atmosphere, is calculated by taking the difference between ADRF at the TOA and ADRF at the surface.

### GDAS

We used potential temperature at the free troposphere pressure level (i.e. 700 hPa) and the surface to calculate lower tropospheric stability (LTS) using the NOAA-NCEP (National Oceanic and Atmospheric Administration National Centers for Environmental Prediction) Global Data Assimilation System (GDAS) assimilated meteorological datasets^67^. The variables are available at 1° × 1° spatial resolution with 21 vertical levels (1000 hPa −100 hPa) at synoptic hours. LTS is calculated as; LTS = θ_700_ − θ_surface_.

## Supplementary information

Supplementary Info

## Data Availability

Satellite (MODIS-AQUA and AURA-OMI) and model reanalysis (MERRA-2) datasets are freely accessible to the public from irrespective websites (Refer to Table S1 in the Supplementary Material).
