# Supplementary material for: Recent Increase in Winter Hazy Days over Central India and the Arabian Sea
Source: Sci Rep. 2019 Nov 22;9:17406. doi: 10.1038/s41598-019-53630-3 (PMC6874585; doi:10.1038/s41598-019-53630-3)
Supplement: Supplementary file 1 — Supplementary Info [file 41598_2019_53630_MOESM1_ESM.docx]

**Supporting Information for**

**Recent Increase in Winter Hazy Days over Central India and the Arabian Sea**

**AbinThomas^1^, Chandan Sarangi^2,*^ and Vijay P. Kanawade^1,*^**

^1^Centre for Earth, Ocean and Atmospheric Sciences, University of Hyderabad, Hyderabad, Telangana, 500046, India

^2^Pacific Northwest National Laboratory, Richland, Washington, 99352, USA

# Table S1. Summary of the satellite and model reanalysis datasets used in this study.

| Data Source and Version | Parameter | Temporal resolution | Spatial resolution | Data Access Link |
| --- | --- | --- | --- | --- |
| MODIS, AQUAL3, C6.1 | AOD | Daily,13:30 | 1º×1º | <https://ladsweb.modaps.eosdis.nasa.gov> |
| AURA OMI, V3 | UV - Aerosol Index | Daily,13:30 | 1º×1º | <https://disc.gsfc.nasa.gov/> |
| MODIS,AQUA L2, C6.1 | Fire counts | Daily,13:30 | 1 km×1 km | <https://ladsweb.modaps.eosdis.nasa.gov> |
| MERRA-2 | AOD and SW fluxes | Hourly, 11:00-14:00 | 0.5º×0.625º | <https://disc.gsfc.nasa.gov/> |
| NOAA-NCEP GDAS | Wind fields and Temp. | 6-hourly, 11:30 | 1º×1º | https://ladsweb.modaps.eosdis.nasa.gov/ |

**Table S2.** Mean difference in ADRF (W/m^2^) between the recent and the past years over study regions.

|  | IGP | CI | AS | BoB |
| --- | --- | --- | --- | --- |
| SWDRF_ATM_ | 2.01 | 4.50 | 3.67 | 0.48 |
| SWDRF_SFC_ | -6.96 | -9.44 | -8.24 | -3.08 |
| SWDRF_TOA_ | -4.95 | -4.94 | -4.57 | -2.60 |


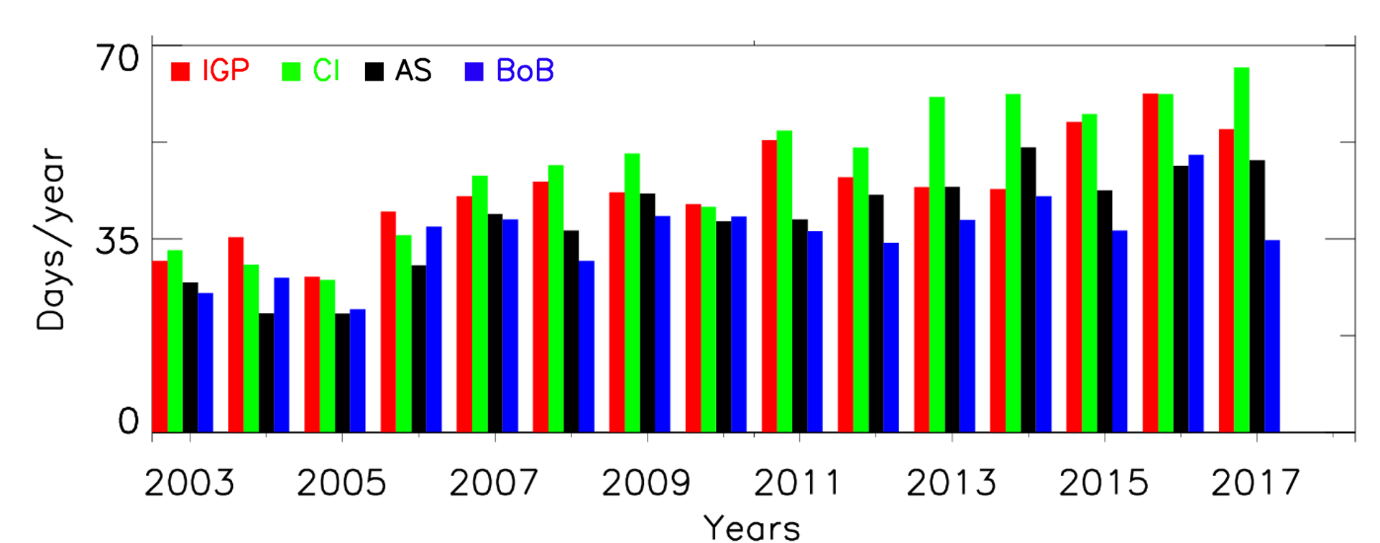


**Figure S1.** Timeseries of the averaged number of days with AOD greater than 66^th^ percentile value from 2003-2017for all study regions.


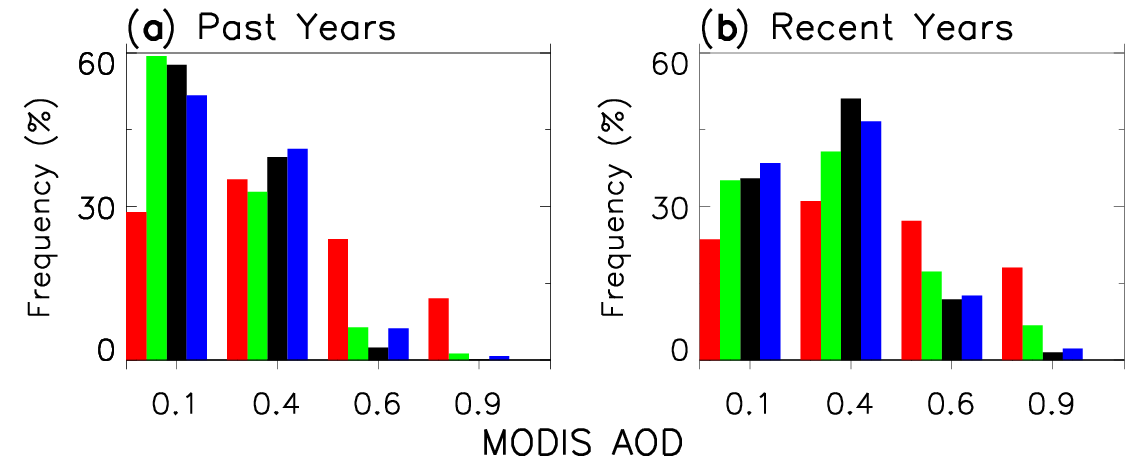

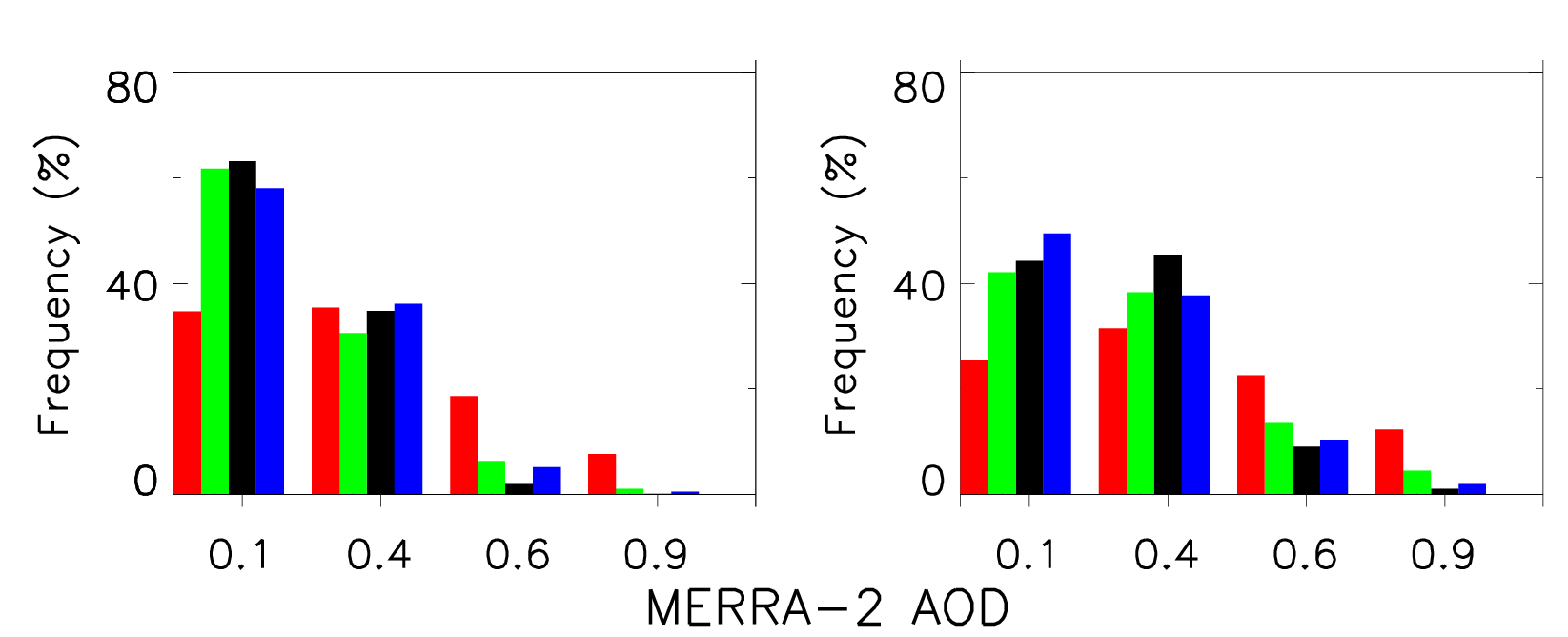


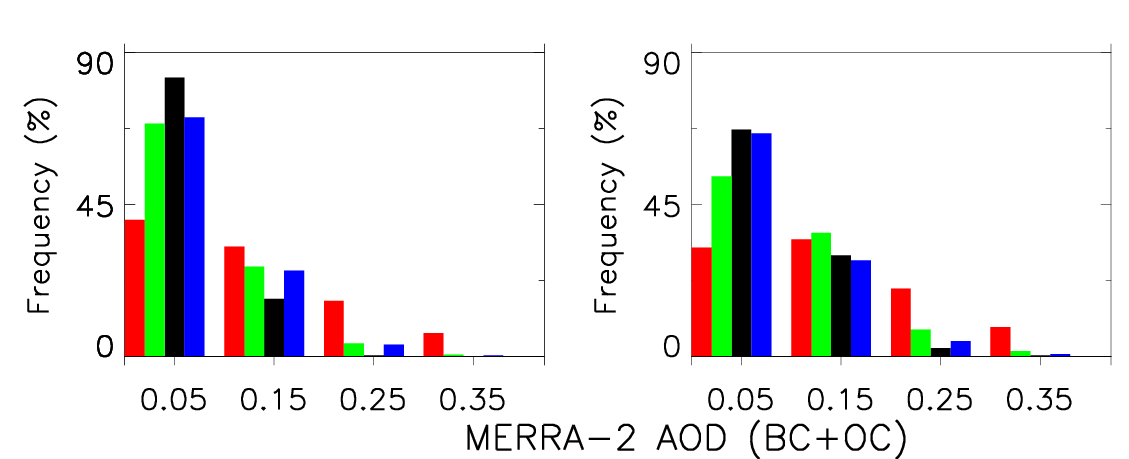

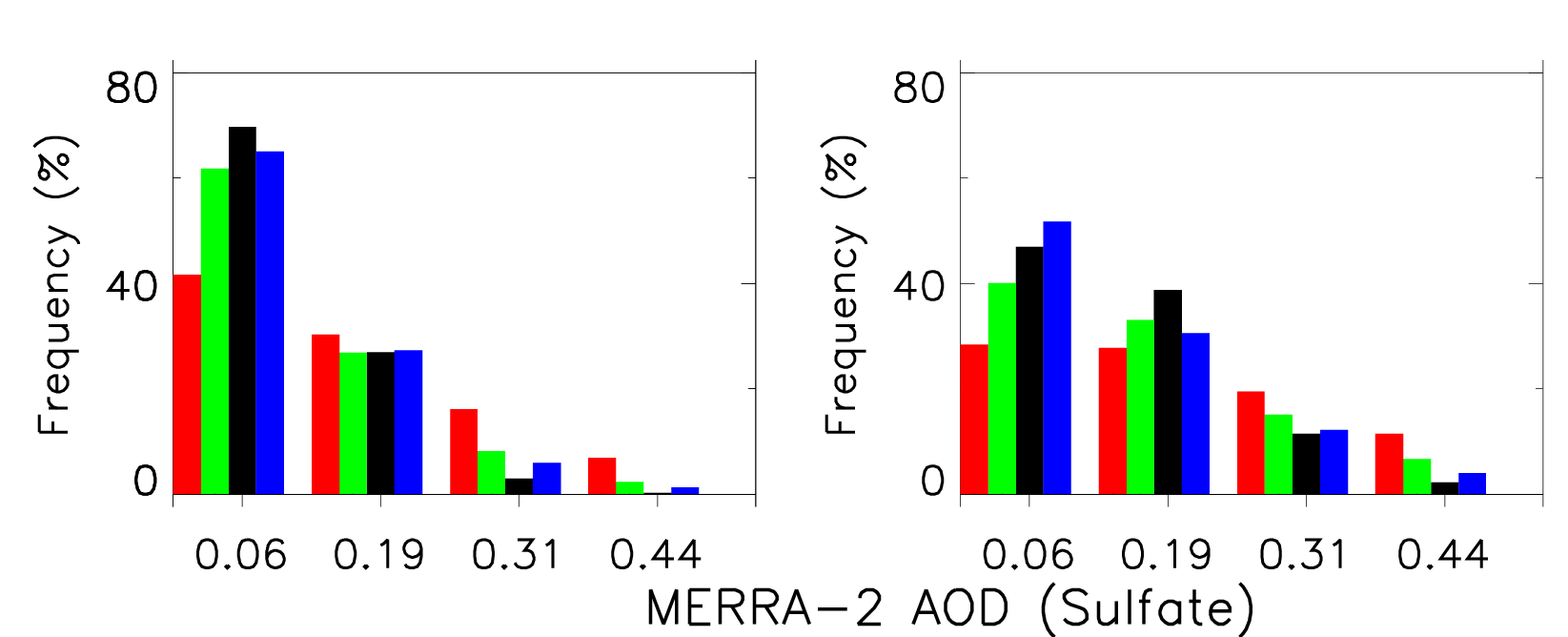

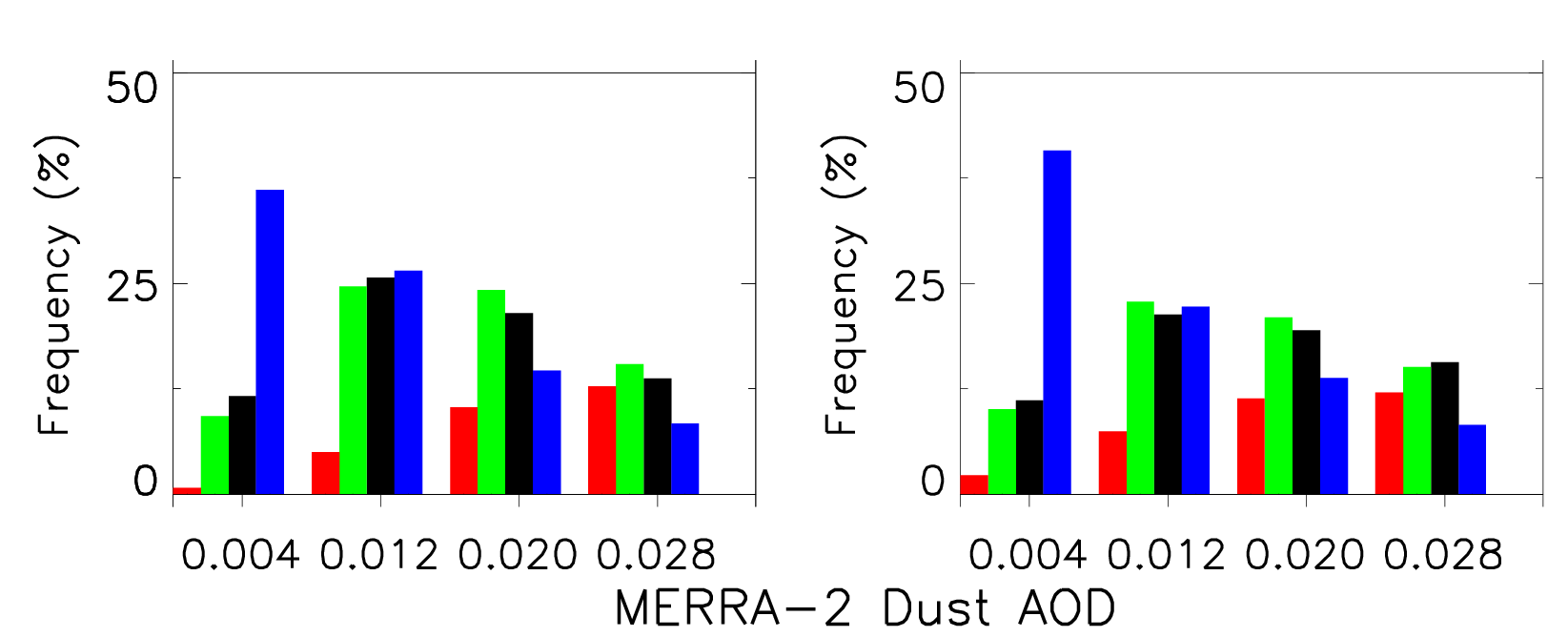

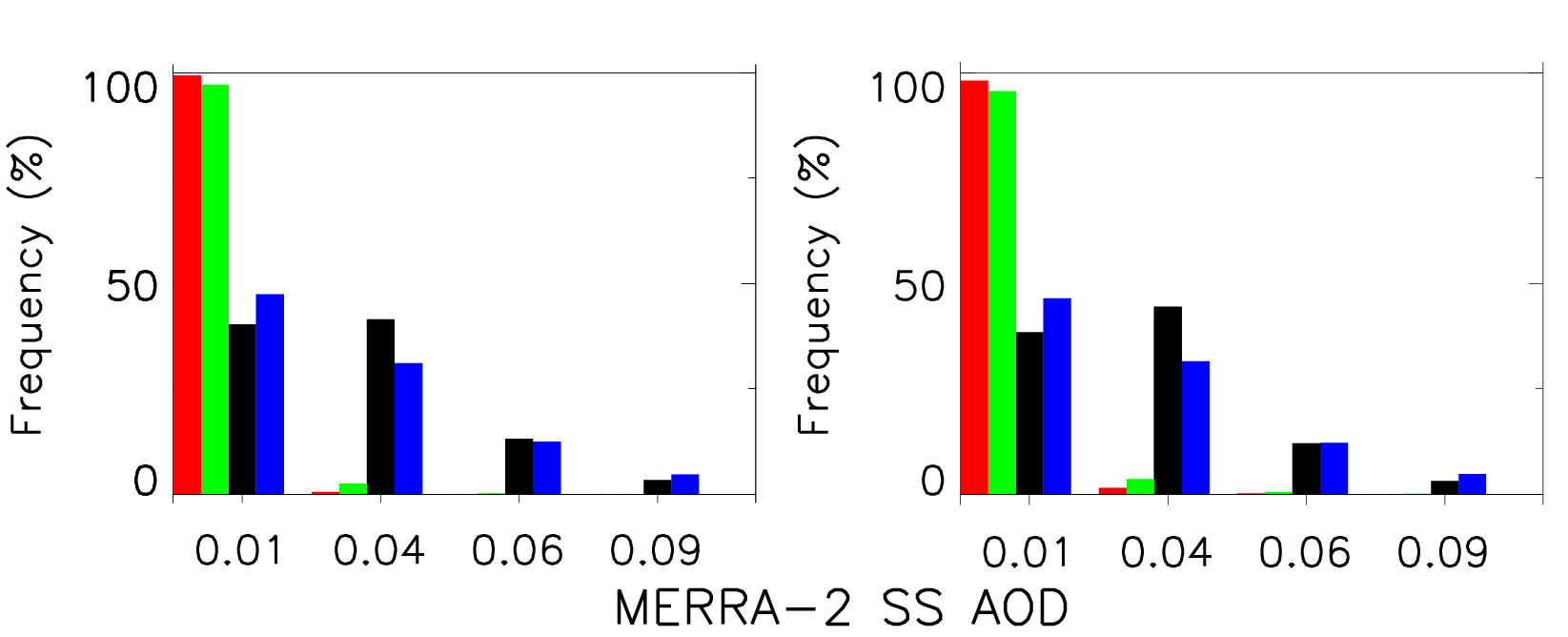
**Figure S2.** The percentage frequency distribution of MODIS AOD and MERRA-2 composite and speciated AOD (total, BC+OC, sulfate, dust and seasalt) over study regions for the past (2003-2007) and the recent (2013-2017) years. Refer to Fig. S1 legend for color coding.


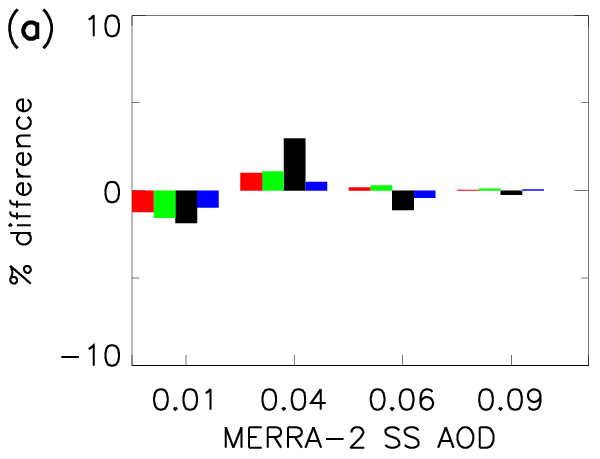

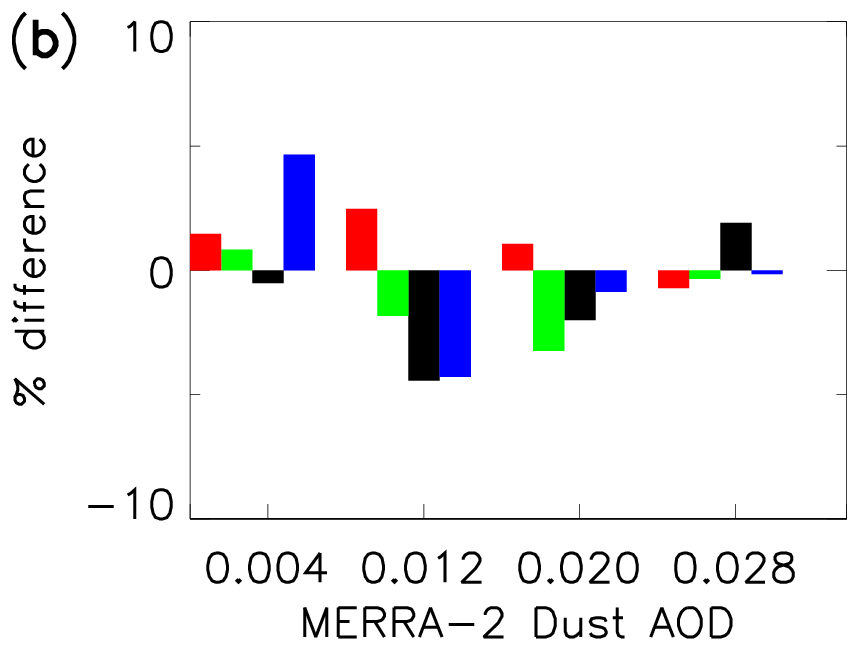
**Figure S3.**Percentage difference in the frequency distribution of MERRA-2 (**a**) Sea Salt and (**b**) Dust AOD between the recent and the past years over study regions. Refer to Fig. S1 legend for color coding.


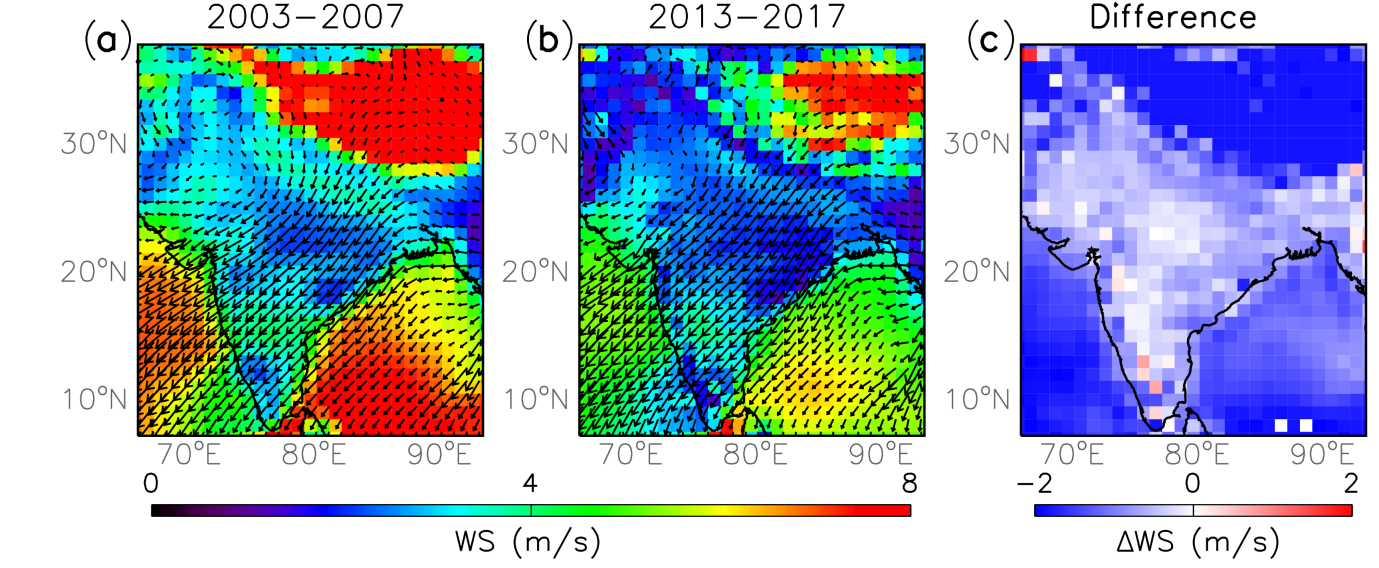
**Figure S4.** The wind speed and vectors at 950hPa level during the past (a) and the recent (b) time periods. (c) The difference in wind speed between the recent and the past years.


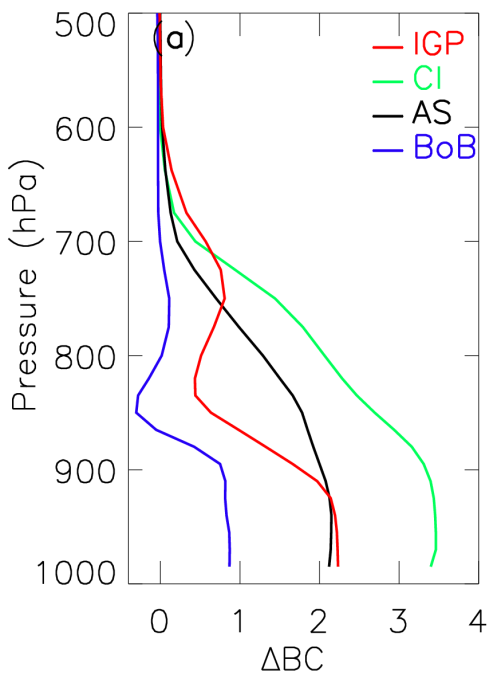

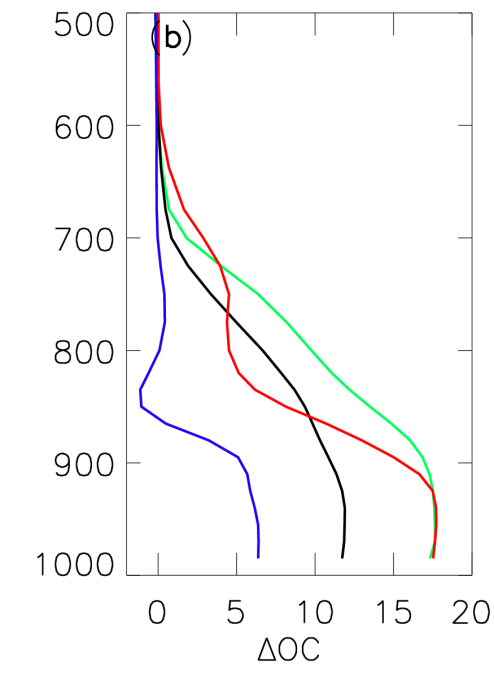

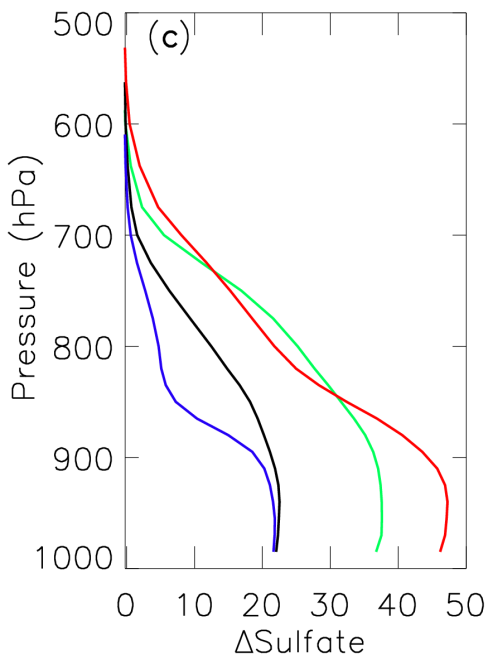
**Figure S5.** MERRA-2 simulated differences in the mixing ratio (in 10^-10^ kg/kg) of (**a**) BC, (**b**) OC and (**c**) sulfate between the past and the recent years during Noverber-February, 2003-2017.


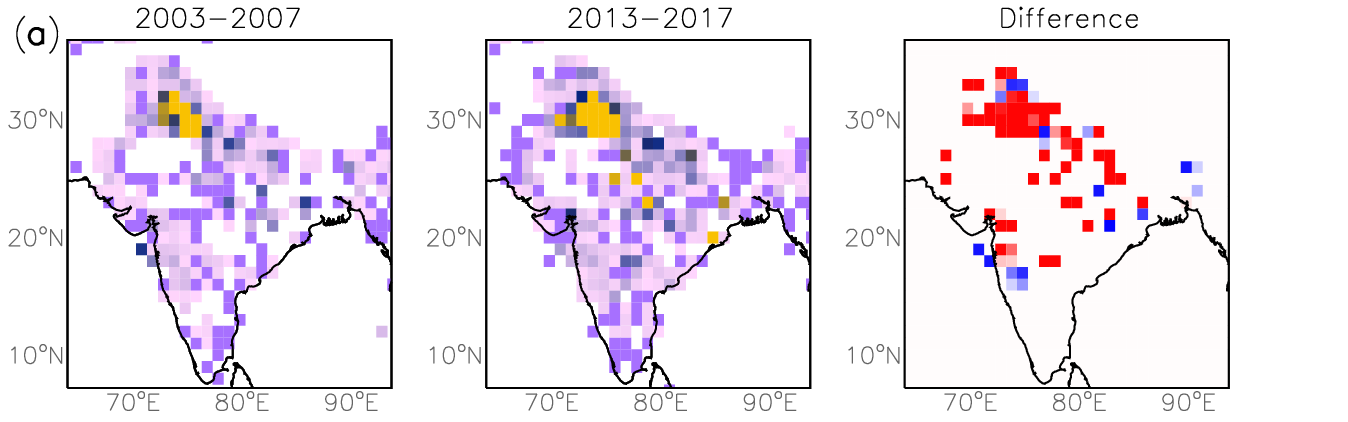


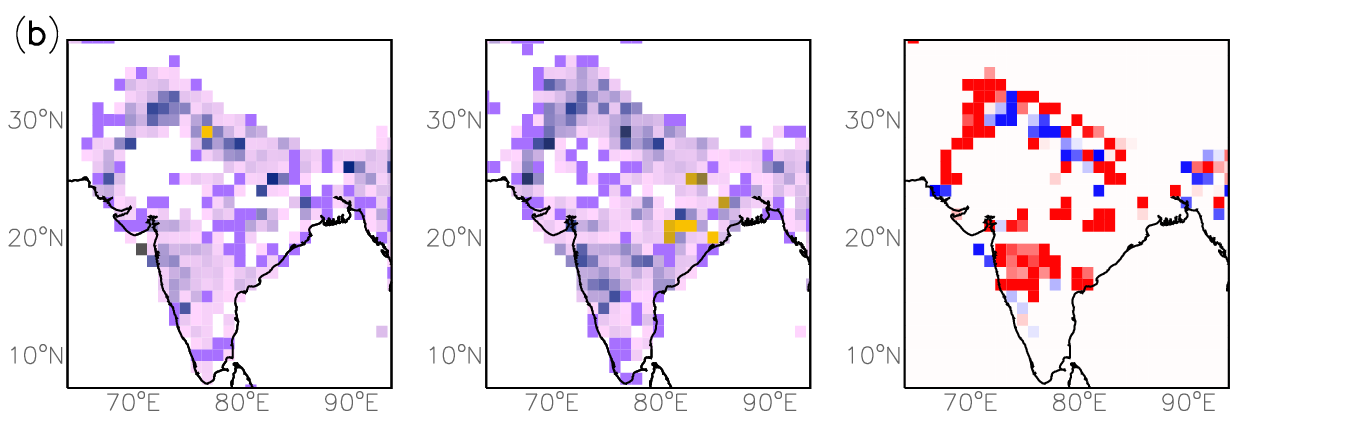


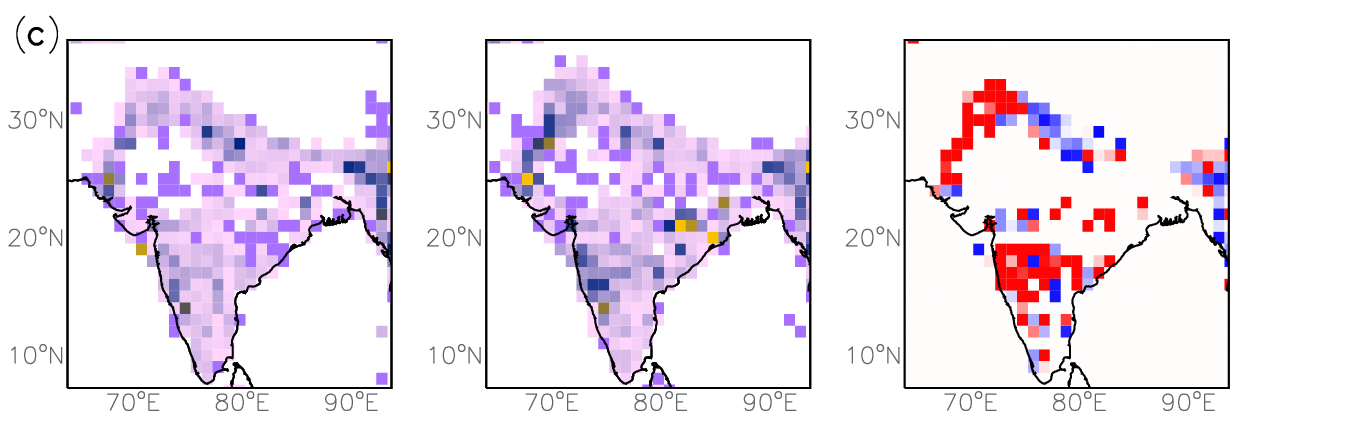


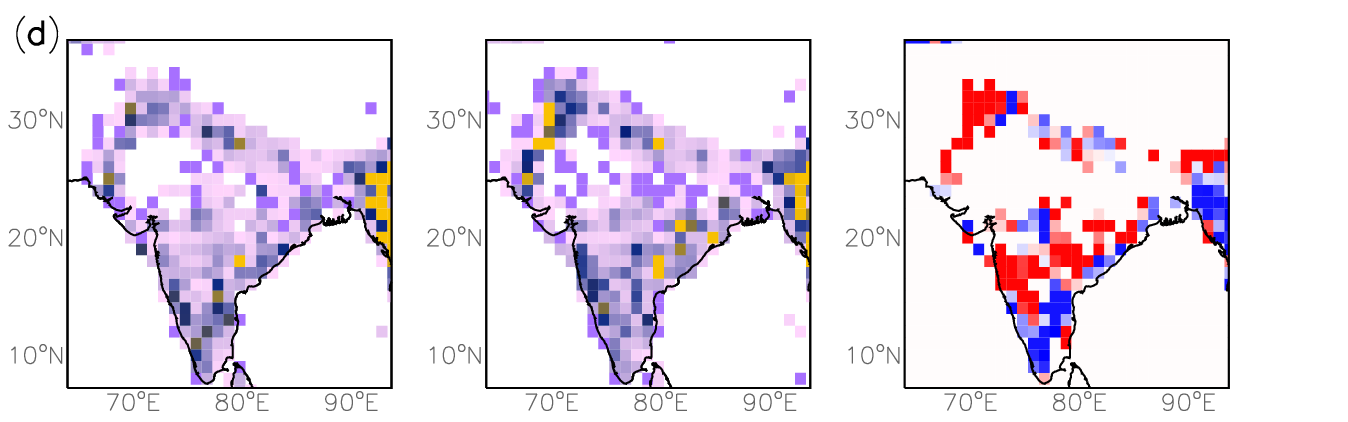

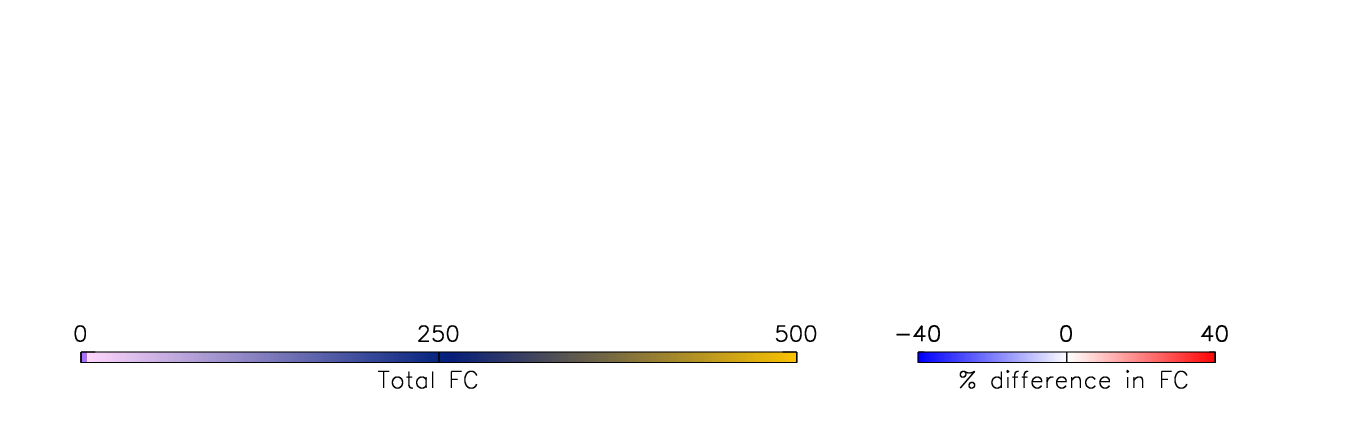
**Figure S6.**Monthly1^o^×1^o^ gridded total fire counts (FC)for the past years (2003-2007), the recent years (2013-2017) and the difference between the past and the recent years for the month of (a)November, (b) December, (c) January and(d) February.


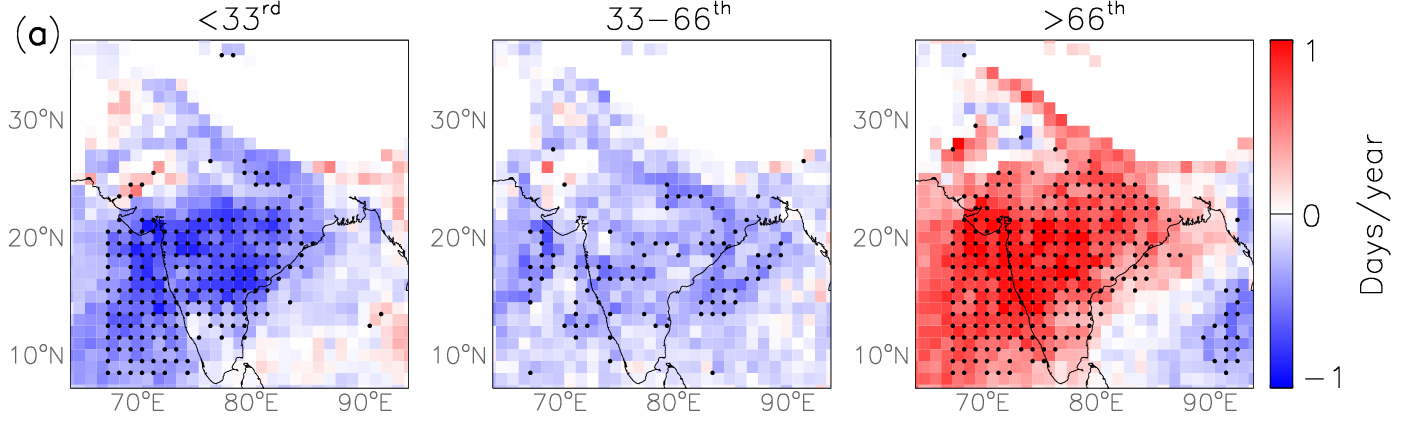

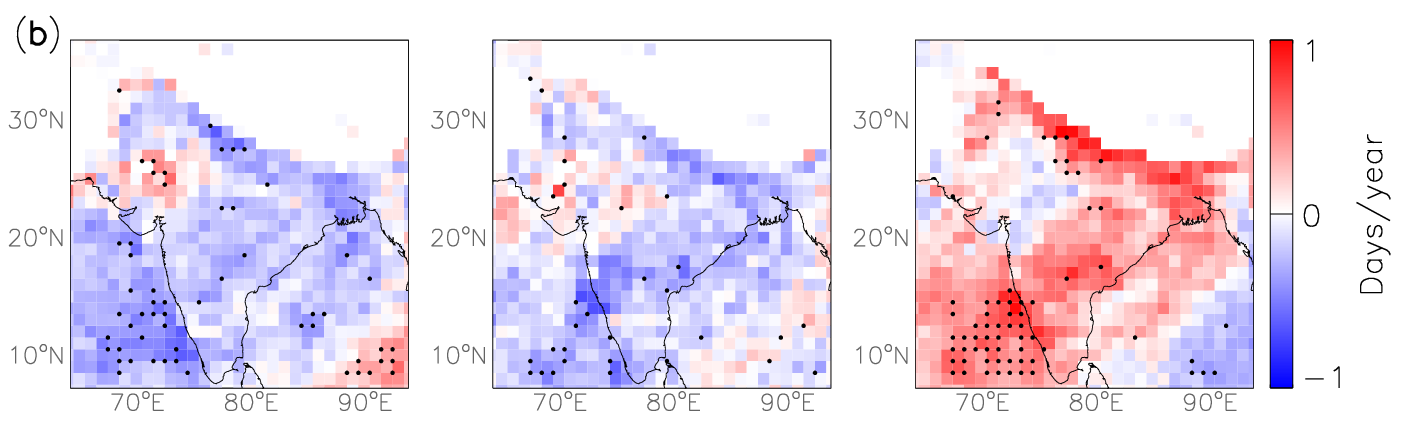

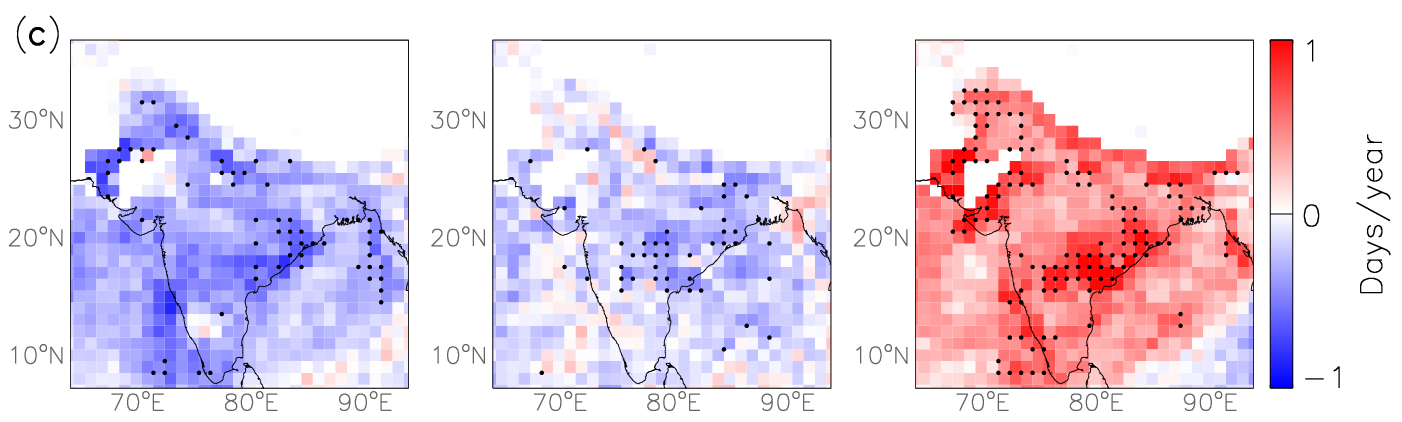

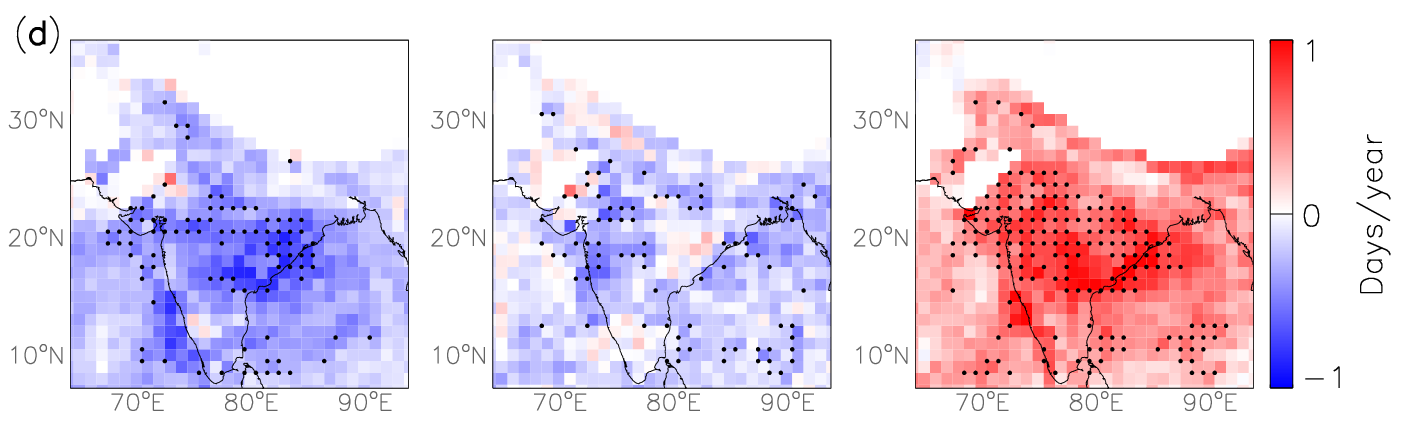


**Figure S7.**Monthly trend in the number of days with <33^rd^, 33-66^th^ and >66^th^ percentile values of AOD for 2003-2017 for the month of (a) November, (b) December, (c) January and (d) February. Black dots indicate statistical significance using Student’s t-test at a confidence interval of 95%.


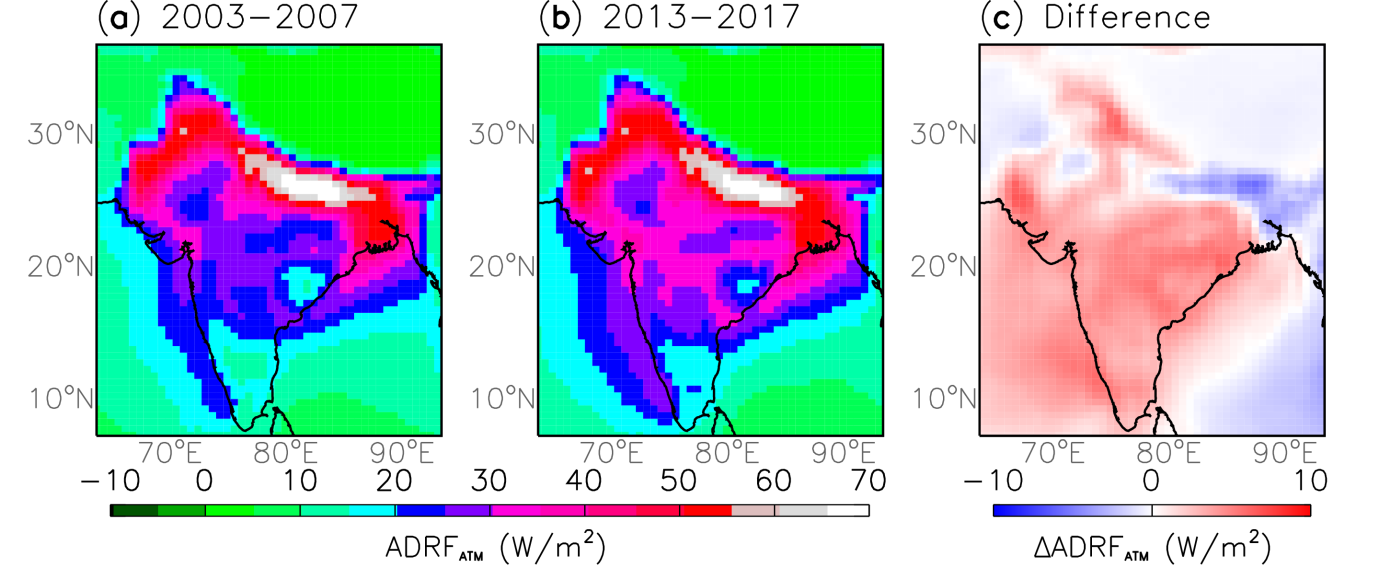


**Figure S8** Averaged ADRF on the atmosphere during (a) past (2003-2007), (b) recent (2013-17) and its (c) difference using MERRA-2 reanalysis.


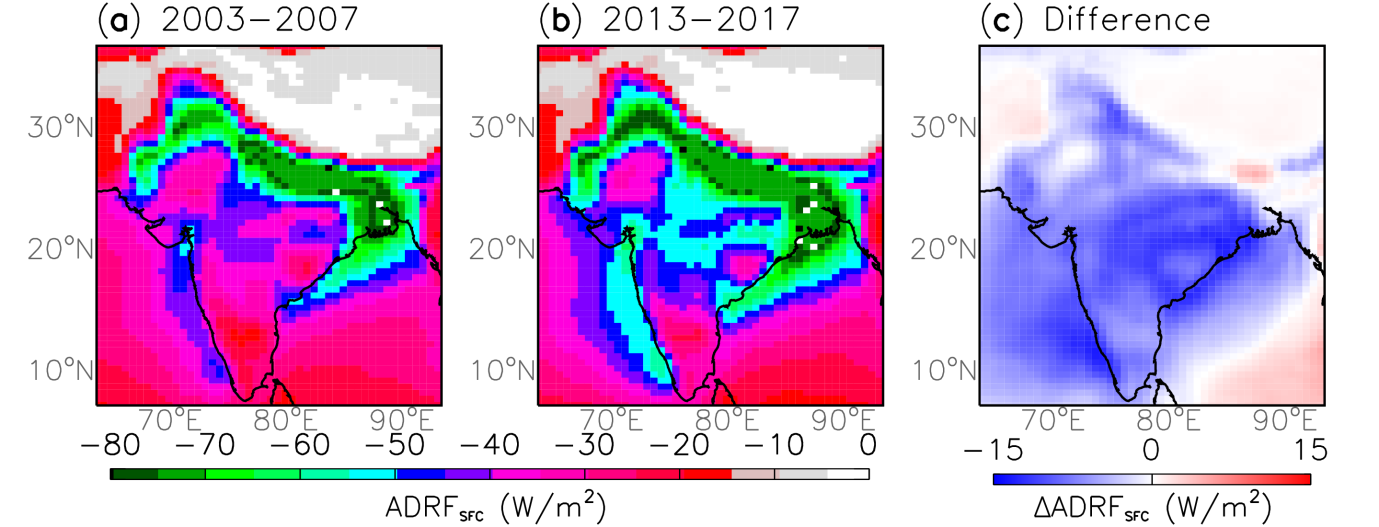
**Figure S9**. Same as Figure S8, but for ADRF on the surface.


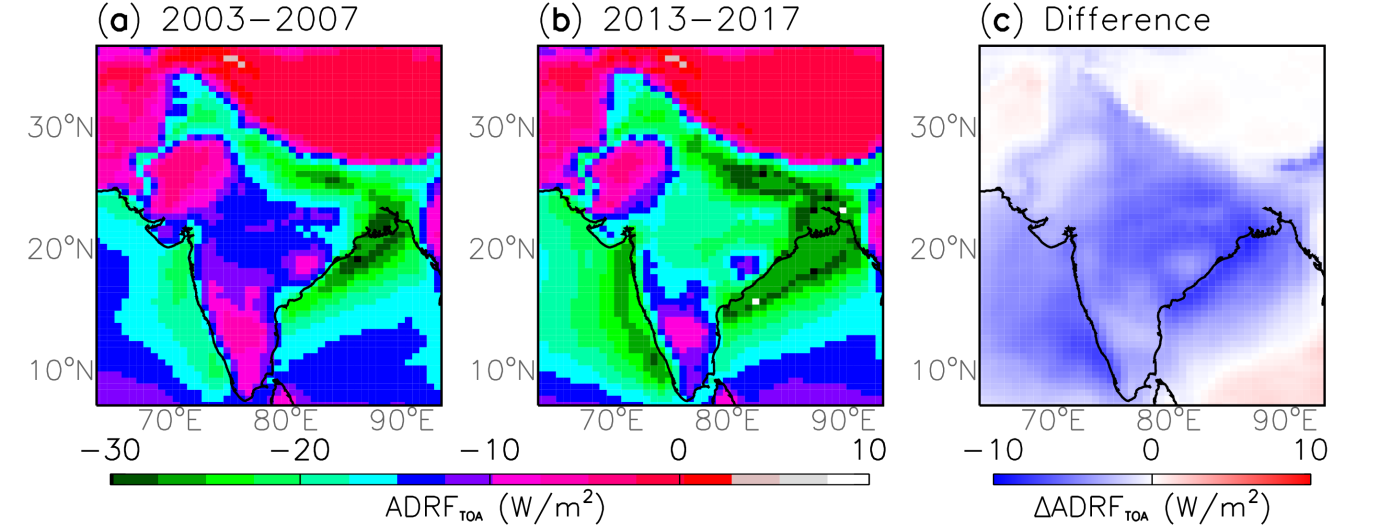
**Figure S10**. Same as Figure S8, but for ADRF on the TOA.

**
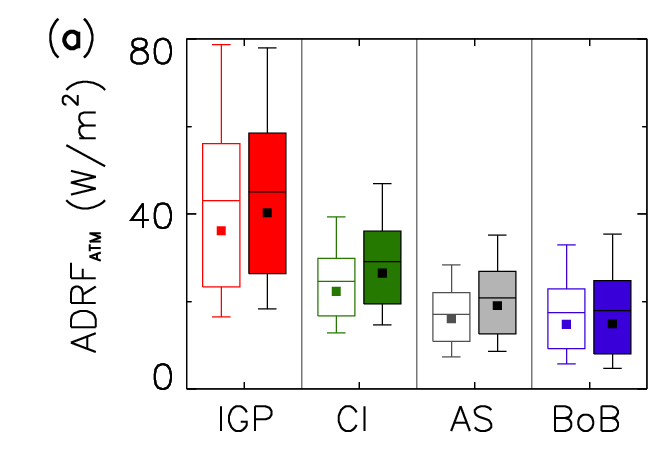

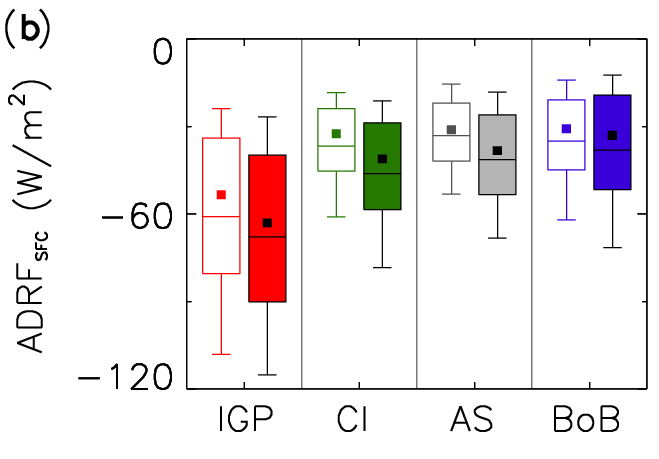

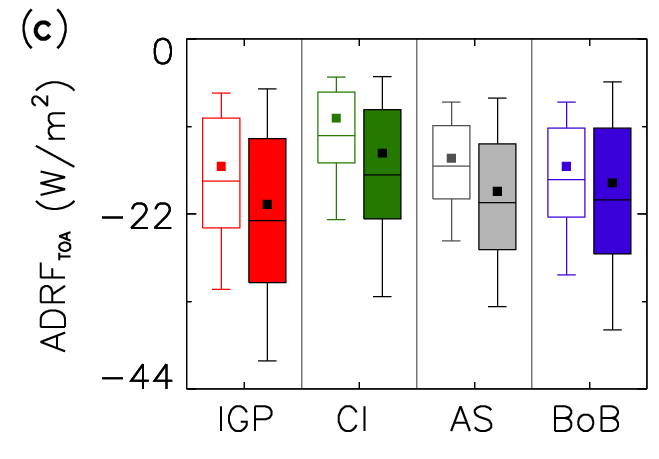
**

**Figure S11.**The aerosol direct radiative forcing (W/m^2^) for the past (hollow box) and recent (filled box) years over the four regions calculated from MERRA-2. The horizontal solid line indicates the mean, small square box indicates the median, upper and lower boundary of the box indicate 25^th^ and 75^th^ percentile values and the whiskers indicate 10^th^ and 90^th^ percentile values.


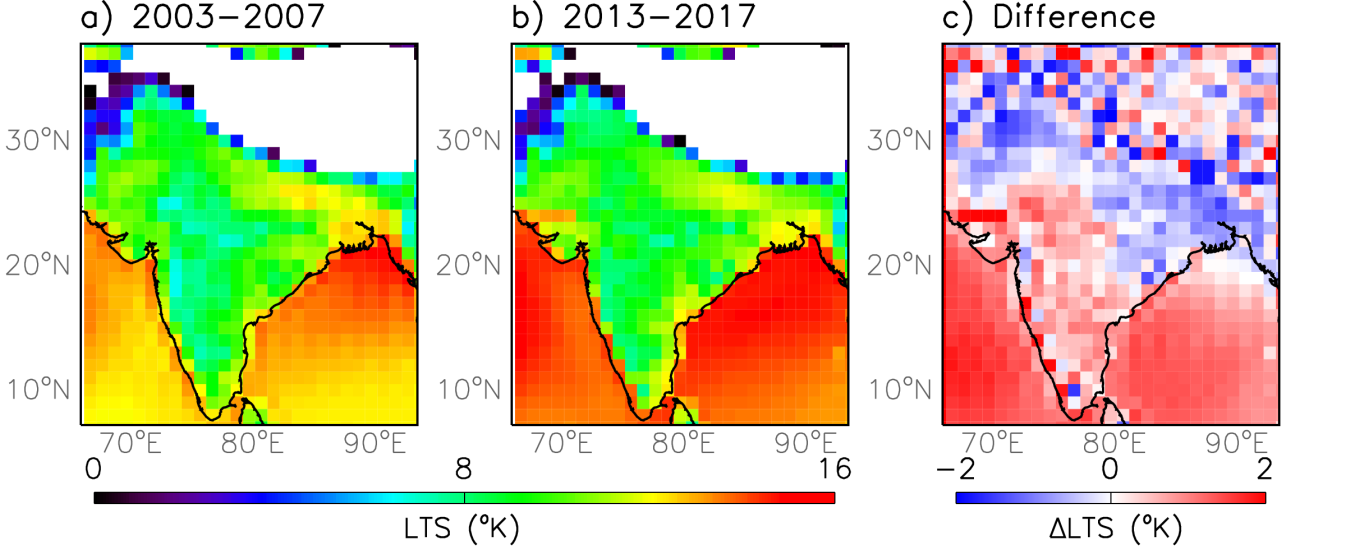
**Figure S12.**Lower tropospheric stability of (**a**) past, (**b**) recent and (**c**) difference between recent and past years using GDAS temperature data.


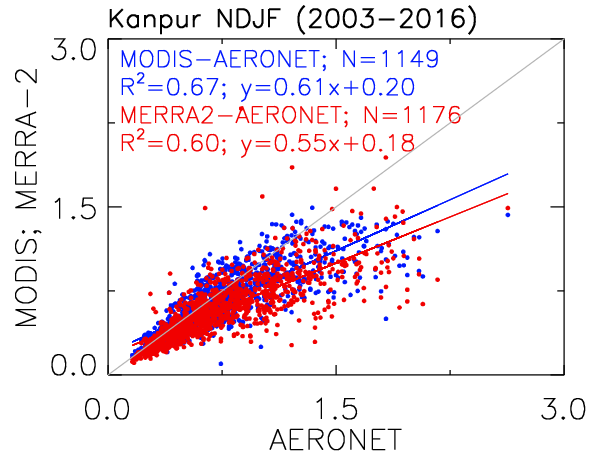

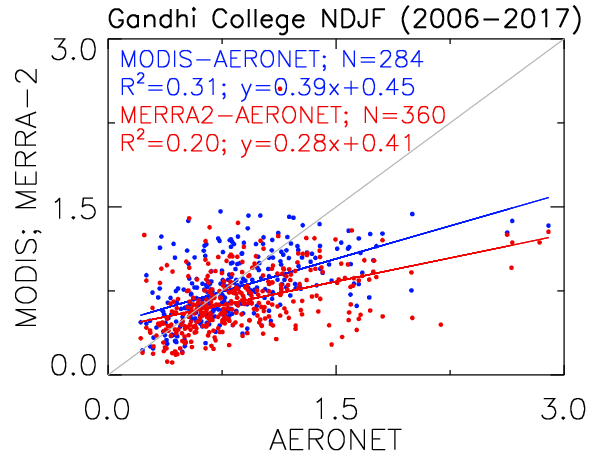

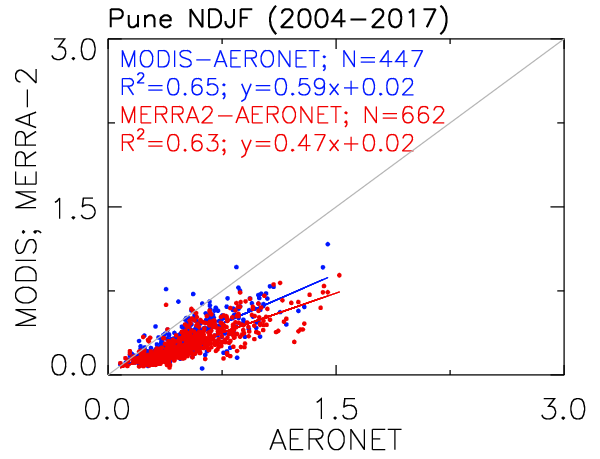

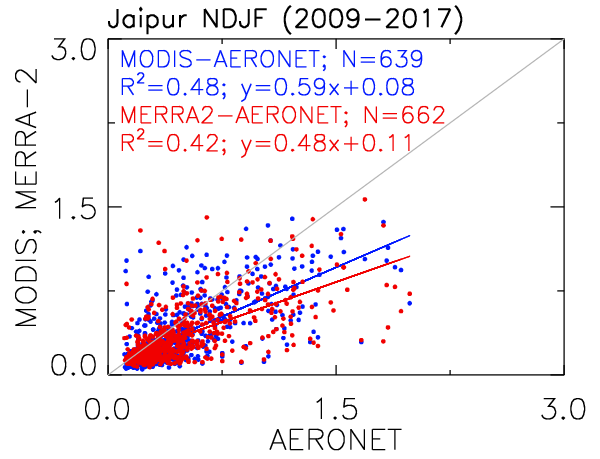


**Figure S13.** Correlation of MODIS-AQUA (blue dots) and MERRA-2 (red dots) AOD with AERONET AOD at four sites over India (Kanpur, Gandhi College, Pune and Jaipur) for the dry winter season (November through February). The dashed line is the 1:1 line. Here, we have used MODIS level-2 Dark Target Deep Blue Combined AOD of collection 6.1 at 0.5µm at a grid resolution of 10km×10km. The MERRA-2 AOD is available at a grid resolution of 0.5^o^×0.625^o^. The MODIS and MERRA-2 datasets are collocated in a 2^o^×2^o^ grid box centred over the AERONET site and then correlated. Overall, MODIS-AQUA AOD is underestimated by a factor of less than 1.6 whereas MERRA-2 AOD is underestimated by a factor of nearly less than 2.
